# PRRSV-1 outbreak in a farrowing farm caused by a vaccine derived strain: a case report

**DOI:** 10.1186/s40813-025-00425-w

**Published:** 2025-02-17

**Authors:** Arnaud Lebret, Patricia Renson, Mathieu Brissonnier, Céline Chevance, Valérie Normand, Justine Favrel, Jean-François Da-Costa, Justine Jeusselin, Théo Nicolazo, Yannick Blanchard, Olivier Bourry, Gwenaël Boulbria

**Affiliations:** 1 PORC.SPECTIVE, ZA de Gohélève, Noyal-Pontivy, 56920 France; 2 REZOOLUTION, ZA de Gohélève, Noyal-Pontivy, 56920 France; 3 ANSES, Ploufragan-Plouzané Niort Laboratory, Zoopôle, BP53, Ploufragan, 22440 France

**Keywords:** Swine, PRRSV, Modified-live vaccine, Sequencing, Production losses, Reversion, Mutation

## Abstract

**Background:**

The benefits of porcine reproductive and respiratory syndrome (PRRS) modified live virus vaccines (MLV) have been largely proven, however, the safety of these vaccines is questioned since vaccine strains can revert to virulence due to random mutations or recombination events. Reversion to virulence has been previously described for PRRSV-2 MLVs and recently for PRRSV-1 MLV after recombination. This case report describes the introduction of a PRRSV-1 strain derived from a MLV associated with an outbreak of reproductive disorder in a 1000-sow farrow-to-wean farm in France.

**Case presentation:**

In January 2023, unusual fever and lethargy in sows, and premature farrowings were reported in a farm that was regularly controlled as PRRS stable, through mass vaccination of the sows. PRRSV-1 was detected by PCR in sows and suckling piglet samples. Sequencing of ORF5, ORF7, and whole genome (WGS) was performed. Time-to-baseline production and total production losses were calculated using statistical process control methods. ORF5 and ORF7 nucleotide sequences indicated that the strain isolated from the clinical samples was differentiable from the DV MLV strain used in the farm (94.1% and 95.9% respectively) but closely related to the VP-046 Bis MLV strain which was never used (99.0% and 99.2% respectively). WGS of the farm PRRSV strain confirmed the high nucleotide identity percentage with the VP-046 Bis MLV strain (98.6%) over the entire genome and no recombination events was detected with MLV strains authorized in France. After different investigations aiming to identify the source of contamination, we were able to detect a closely related strain (99.46% of identity with the case farm strain across the entire genome) in a wean-to-finish farm located 400 m further. It took 17 batches (34 weeks) to recover the baseline production of piglets after implementation of a PRRSV stabilization protocol, which represented a total loss of 812 weaned piglets.

**Conclusion:**

This is the first case report of a PRRSV-1 MLV which might have reverted to virulence in France and has caused substantial economic losses.

## Background

Porcine Reproductive and Respiratory Syndrome Virus (PRRSV) is the aetiologic agent of PRRS, the most economically important disease of the swine industry [1]. The presence of PRRSV can intensify bacterial co-infections and potentially lead to increased antibiotic usage which make PRRS management essential in swine production systems [2, 3]. PRRSV belongs to the Arteriviridae family [4], genus Betaarterivirus, with two species: Betaarterivirus suid 1, also named PRRSV-1, which has a European origin; and PRRSV-2, renamed Betaarterivirus suid 2, originating from North America. Since its emergence in North America in the late 80’s and in Europe a few years later, the virus remains a challenge for pig farmers and practitioners that continuously aim to improve diagnostics and control in pig herds. In France, only closely related strains of PRRSV-1 have been detected and the virus is mainly endemic in Brittany where more than 60% of French pork production is settled [5].

There are a variety of programs implemented for the control of PRRSV. To reduce the clinical impact of the disease during PRRSV outbreaks and/or to tackle the virus circulation, vaccination with modified live virus vaccines (MLVs) is quite common [5]. MLV1 against PRRSV-1 and MLV2 against PRRSV-2 could be used especially in breeding herds. In France, at this time, five MLV1s are on the market: Porcilis^®^ PRRS (strain DV) from Intervet (Beaucouzé, France) which was the first one to be authorized in the French market, followed by Unistrain^®^ PRRS (strain VP-046 Bis) from Hipra (Amer, Spain), ReproCyc^®^ PRRS EU for the gilts and sows and Ingelvac^®^ PRRSFLEX EU for weaned pigs (strain 94881) from Boehringer Ingelheim Animal Health (Lyon, France) and the last one, Suvaxyn^®^ PRRS MLV (strain 96V198) from Zoetis (Zaventem, Belgium). Since PRRSV-2 is absent in France, MLV2s are not authorized in the country.

Even if the benefits of MLVs have been largely proven, the safety of these live vaccines is frequently questioned. Since MLV can return to virulence due to mutations and/or recombination events, many concerns have raised in the last years. Reversions causing clinical diseases, both reproductive and respiratory, and impacting production performances has been previously demonstrated for MLV2 [6–8] and more recently for MLV1 after recombination between different MLV1s [9, 10]. The partial reversion to virulence of the DV strain was also shown after only few passages in pigs following acquisition of specific mutations [11]. This case report describes the clinical situation and outcome in a farm following the introduction of a PRRSV-1 strain derived from a vaccine strain.

## Case presentation

### Farm description

**Fig. 1:**
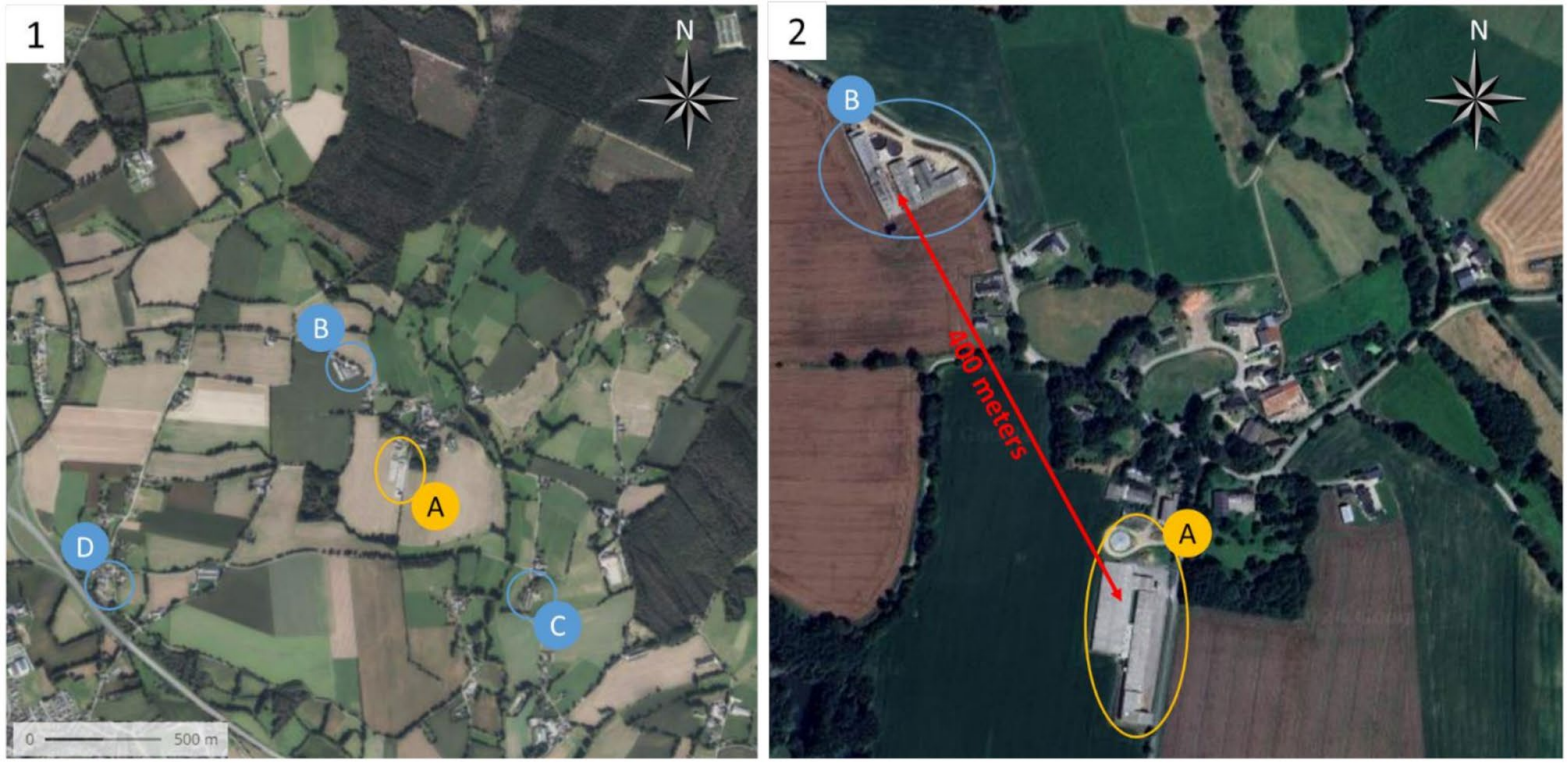
(1) Pig farms located within a two-kilometer radius. (2) The two farms infected by the case vaccine-derived PRRSV strain. (**A**) the case farm; (**B**, **C** and **D**) weaning-to-slaughter farms from unknown PRRSV-status

The farrowing farm is located in Brittany (France), a pig dense area. Three weaning-to-slaughter farms from unknown PRRSV-status are located within a two-kilometer radius (Fig. 1). The nearest neighbouring farm is only 400 m away. The case farm owned 1000 sows conducted in ten batches with farrowings every two weeks. The sow’s replacement is done by purchasing gilts from an outside PRRSV-free nucleus farm with 50 gilts entering in quarantine every four weeks. Inseminations are performed using purchased semen from a PRRSV-free boar stud. In total, the farm has two farrowing rooms (one per batch) of 85 places. After artificial insemination in the breeding unit, sows are kept in a large gestating unit with deep straw bedding. Sows are fed with a commercial diet.

At the moment of the clinical outbreak, gilts and sows were routinely vaccinated against influenza A virus H3N2, H1N1 and H1N2 (two times in quarantine and four weeks before farrowing), *Escherichia coli* and *Clostridium perfringens* type C (two times in quarantine and three weeks before farrowing), porcine circovirus type 2 (two times in quarantine and two weeks before farrowing) and Parvovirus and *Erysipelothrix rhusiopathiae* (two times in quarantine and two weeks after farrowing).

Regarding PRRSV, for the past five years, sows received a MLV1 (Porcilis^®^ PRRS, MSD) twice in quarantine and three times a year (sow mass vaccination every 4 months). The farm was regularly submitted to PRRSV monitoring and classified as PRRSV stable with vaccination according to the classification of the American Association of Swine Veterinarians (AASV) [12, 13]. The level of external and internal biosecurity of the farm was very good according to French regulation.

### Case history

In January 2023, the farmer reported unusual fever, lethargy and premature farrowings in the farrowing batch and asked for a veterinarian visit.

Within two days, five sows out of 85 have farrowed between 109 and 111 days of gestation. Most of the piglets were stillborn and the rest of them were splay-legged. During the farm visit, 10 lethargic sows presented a rectal temperature from 40 °C to 41.2 °C. The other sows of the batch seemed healthy. In the batch that farrowed 10 days before, we also observed five anorexic sows with rectal temperature between 39.8 °C and 40.5 °C. The farrowings on this batch were considered as normal by the farmer. In the insemination and gestation units, no abnormal clinical sign was observed.

Nasal swabs and blood samples from eight sows showing fever and lethargy in the farrowing batch were taken by the veterinarian. Sows nasal swabs were tested individually by PCR for influenza A virus using Adiavet SIV Real Time kit and PRRSV using Adiavet PRRS Real Time kit (Adiagene, BioX Diagnostics, Ploufragan, France). Sows blood samples were tested individually for PRRSV by PCR.

In parallel, 30 piglets before weaning from the previous batch were bled and tested by pool of five for PRRSV by PCR.

All sows present in the two farrowing rooms were treated orally with paracetamol for five days (30 mg/kg body weight/day).

### Laboratory findings

Sow nasal swabs were tested negative for influenza A virus by qPCR but positive in three sows out of eight for PRRSV. Sows blood samples were all positive for PRRSV as well as the six pools out of six of piglets blood.

As the farm was regularly vaccinated with a MLV1 (Porcilis^®^ PRRS, DV strain), ORF7 and ORF5 sequencing were performed on three samples in order to characterize the PRRSV-1 strain detected: two (one nasal swab and one blood sample) from a diseased sow and one from piglets (pool of five blood samples). The sequence comparison between the farm strain and the different MLV1 strains present in the French market are presented in Table 1.

**Table 1:** Percentages of nucleotide identity for ORF5 and ORF7 between the PRRSV-1 farm strain and the different MLV1 strains present in the French market (DV strain was the one used in the farm)

	DV strain (MW674755)		VP-046 Bis strain (GU067771)		94,881 strain (KT988004)		96V198 strain (MK876228)	
	ORF7	ORF5	ORF7	ORF5	ORF7	ORF5	ORF7	ORF5
Farm strain (from sow’s nasal swab)	95.9	94.1	99.2	99.0	94.6	92.4	92.3	89.3
Farm strain (from sow’s blood sample)	95.9	94.1	99.2	99.0	94.6	92.4	92.3	89.3
Farm strain (from piglet pool of 5 blood samples)	95.9	94.1	99.2	99.0	94.6	92.4	92.3	89.3

ORF5 and ORF7 sequencing results indicated that the strain isolated from the clinical samples was differentiable from the one of the vaccine used in the farm but was close to another one from a different vaccine: the Unistrain^®^ PRRS (VP-046 Bis strain). To confirm the ORF5 and ORF7 sequencing results, one of the three samples (one blood sample from the pool of piglet blood samples with the lowest PCR Ct value) was submitted to a whole genome sequencing. First, the farm PRRSV-1 strain was isolated from one piglet blood sample and amplified once on porcine alveolar macrophages. Then, the whole genome sequencing of the isolate was performed by the ANSES Ploufragan Next Generation Sequencing platform on a Ion Torrent Proton sequencer (ThermoFisher Scientific, Waltham, MA, USA) (PRRS-FR-2023-22-10-1 strain: GenBank accession No PQ572682). By Basic Local Aligment Search Tool (BLAST) analysis, the SHE strain (derived from the VP-046 Bis vaccine strain, accession No. GQ461593) was identified as the strain with the highest homology with the farm strain (98.62% of identity). Using the Muscle alignment algorithm, the high homology of the farm strain with the VP-046 Bis strain was confirmed at the full genome level (Table 2). Nevertheless, the 1.4% diversity with the VP-046 Bis strain indicates that the farm strain has clearly evolved compared to the vaccine strain, as variants from vaccine strains exhibited much lower diversity with their parental vaccine strain [11].

**Table 2:** Percentages of nucleotide identity for the full genome between the PRRSV-1 farm strain and the different MLV1 strains present in the French market (DV strain was the one utilized in the farm)

	DV strain (MW674755)	VP-046 Bis strain (GU067771)	94,881 strain (KT988004)	96V198 strain (MK876228)
Farm strain (piglet’s blood sample)	94.1	98.6	88.4	90.2

**Fig. 2:**
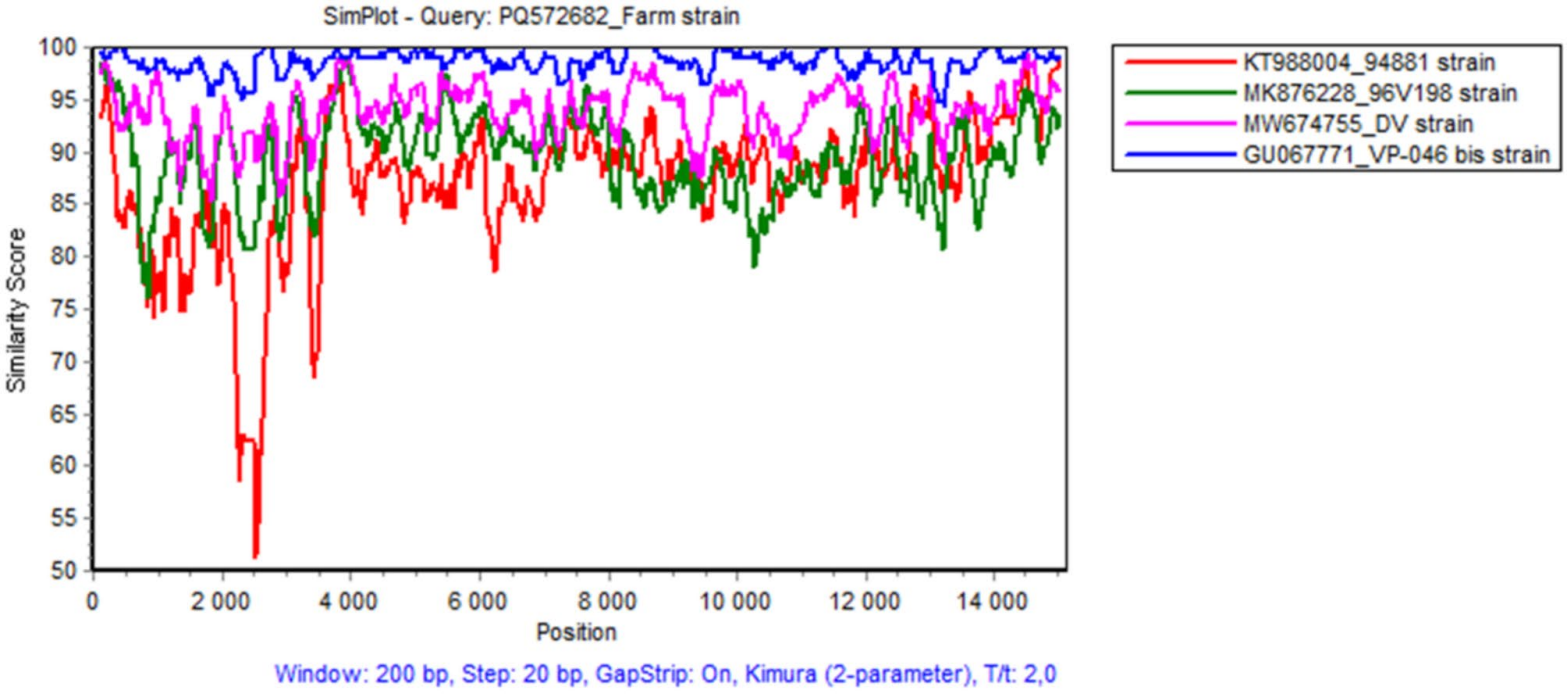
Sequences similarity percentage for the full genome between the farm PRRSV-1 strain and the different MLV1 strains present in the French market visualized with the Simplot program

The similarity study using the Simplot© program (version 3.5.1) [14] showed that the genomic similarity percentages of the farm PRRSV-1 strain with the VP-046 Bis vaccine strain were homogenous over the entire genome and that no recombination events can be detected with MLVs commercially available in France (Fig. 2). These results were further confirmed using a Recombination Detection Program (RDP4 version; [15]) analysis using full genome (public and non-public) sequences from 60 PRRSV-1 strains (including the MLV1s available in France and 26 PRRSV-1 field stains from France). The RPD4 results did not identify the farm strain as a recombinant strain (data not shown). However, due to the limited number of PRRSV-1 field strains included in the analysis, recombination events with unknown PRRSV-1 field strains could not be completely excluded.

In order to further identify the location of the divergence observed between the farm strain and the VP-046 Bis strain, percentages of identity were calculated at the nucleotide level for each Open Reading Frame (ORF) and at the amino acid level for each related produced polyprotein or protein using the Muscle and the Clustal omega alignment algorithms respectively (Table 3). The results showed that the nucleotide diversity in the coding regions (98.55%) was conserved at the protein level with 98.4% of amino acid identity across all protein sequences. Some proteins were well conserved like the structural M and N proteins (99.42% and 100% respectively), as well the polyprotein pp1b (99.11%). Higher genetic diversity that could impact protein functionality was found in GP2, E, GP3 and GP4 with percentages of amino acid identity lower than 98% but never below 96% compared with the VP-046 Bis strain.

**Table 3:** Percentages of nucleotide or amino acid identity between the PRRSV-1 farm strain and the VP-046 bis strain for each PRRSV-1 ORF or related protein

ORF / produced protein	% nucleotide identity (number of mutated nt out of the total ORF nt)	% amino acid identity (number of mutated aa out of the total protein aa)
ORF1a / pp1a	98.48% (109/7191)	98.29% (41/2396)
ORF1b / pp1b	98.84% (51/4392)	99.11% (13/1463)
ORF2a / GP2	98.40% (12/750)	97.59% (6/249)
ORF2b / E	97.65% (5/213)	97.14% (2/70)
ORF3 / GP3	97.87% (17/798)	96.98% (8/265)
ORF4 / GP4	97.28% (15/552)	96.17% (7/183)
ORF5 / GP5	99.01% (6/606)	98.01% (4/201)
ORF6 / M	99.04% (5/522)	99.42% (1/173)
ORF7 / N	99.22% (3/387)	100% (0/128)
All ORFs / proteins	98.55% (223/15411)	98.40% (82/5128)

As MLV1 strains replicates in MARC-145 cell line but not in primary alveolar macrophages (PAMs) and PRRSV-1 field strains replicates in PAMs but not in MARC-145 cells, we compared the viral replication of the farm strain by titrating the isolate in both cell types. The results showed that the farm strain is still able to replicate in MARC-145 cells but with lower titer than in PAMs (10^5.6^ TCID50/ml and 10^7.2^ TCID50/ml respectively), illustrating the adaptive evolution of this MLV-derived strain toward a re-adaptation to the natural target cells of the virus.

Taking into account the different laboratory results obtained, we concluded that the PRRSV-1 strain circulating in the farm and associated with the disease was a non-recombinant strain of MLVs commercialized in France, derived from the VP-046 Bis vaccine strain and showing a significant evolution (1.4% divergence) compared to the parental strain.

On the same time, we were able to discuss with the veterinarian and the owner of the nearest farm (farm B in Fig. 1) and they provided us blood samples of growing pigs of 16 weeks of age. From these samples, a PRRSV-1 strain was detected by qPCR and whole genome sequencing was possible (PRRS-FR-2023-22-09-1 strain: GenBank accession No PQ572681) showing a 99.46% similarity with the case farm strain (data not shown).

### Control measures implemented

Following these results, the farm was closed and no gilts were introduced during 12 weeks. The entire sow herd was mass vaccinated two times at four weeks interval (weeks 6 and 10 in 2023) with the MLV1 previously used in the farm (Porcilis^®^ PRRS). In week 20/2023, we collected blood samples on 30 due to wean piglets. The samples were pooled by five and submitted for PRRSV PCR. One pool out of six returned positive (Ct = 31) but the laboratory was not able to sequence ORF5 and ORF7. Two weeks after, the same sampling procedure and analysis was performed and four pools out of six returned positive. ORF5 and ORF7 were sequenced and showed 100% similarity for both ORFs to the PRRSV-1 strain isolated in January.

In view of these results, we did not change the vaccination scheme but strongly reviewed with the farmer the management of sows and piglets in farrowing rooms according to Management Changes to Reduce Exposure to Bacteria and Eliminate Losses (Mac REBEL™) strategy applied to PRRS during 6 months [16]. Briefly, the following biomanagement measures were applied: no longer use of nurse sows, cross-fostering within 48 h post farrowing, unidirectional human flow from youngest to oldest piglets, no longer use of processing carts for piglets. Other measures such as changing needles and blades between litters, changing needles between sows, all-in/all-out in farrowing rooms and strict cleaning and disinfection procedures were already in place.

### Outcome of the case

In January 2024, blood samples on due-to-wean piglets according to AASV classification allowed us to classify the farm as stable with vaccination, so approximatively one year after the outbreak. Briefly, between November 2023 and January 2024, we sampled 60 due-to-wean piglets (one per litter) per batch on four successive batches and tested them (pooled by five) by qPCR. All the results were negative.

To estimate the losses due to the PRRSV outbreak, the time-to-baseline production (TTBP) was calculated. It is defined using statistical process control methods to represent time to recover the number of pigs weaned per week that the herd had prior to PRRSV detection [17]. An exponentially weighted moving average (EWMA) was performed as the statistical process control tool to establish the control limits, and this analysis was conducted using Minitab software (Minitab Statistical Software 22). The data from a production cycle (10 batches in our case) prior to PRRS outbreak were used as BASELINE data. A kappa (weight) of 0.400 and 3-sigma were applied to define the control limits as previously described [18].

**Fig. 3:**
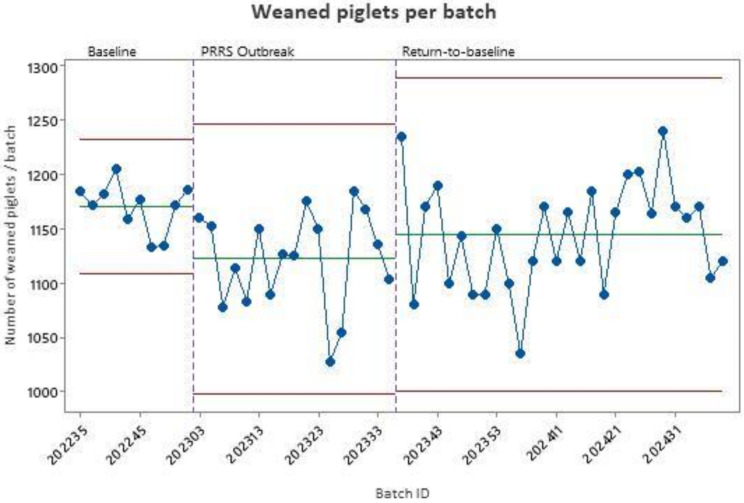
Total weaned piglets per batch monitoring: X-bar control chart before and after PRRS outbreak

As presented in Fig. 3, it took 17 batches (34 weeks) to recover the baseline production of piglets per batch resulting in a total loss of 812 piglets.

## Discussion and conclusion

This case report describes the clinical and virological aspects of a PRRS outbreak in a previously stable breeding herd following the introduction of a virus strain derived from a vaccine strain. Reversion to virulence of vaccine strains has been previously described mainly for PRRSV-2 strains. For example, in Denmark in the late 90’s, the implementation of a national PRRS control program using the Ingelvac PRRS MLV (Boehringer Ingelheim, Ingelheim, Germany) has led to the spread of this MLV2 strain from vaccinated growing pigs to non-vaccinated sows which then experienced reproductive failures [19]. This MLV2 strain not only spread within the vaccinated swine herds but also spread from vaccinated to non-vaccinated herds. Further genetic analysis showed that this reversion to virulence was associated with specific mutations that reverted to the genome of the virulent parental strain of the MLV2 [6]. To the best of the author’s knowledge, this is the first time such an outbreak has been linked to infection with a PRRSV-1 vaccine-derived strain. Nevertheless, partial reversion to virulence of a MLV1 strain has already been described under experimental conditions. In 2021, Eclercy and al. showed that the re-adaptation of the DV vaccine strain to pigs was associated with faster replication and increased transmission rate, suggesting a partial reversion to virulence [11]. At the genetic level, the authors identified three mutations linked to pig re-adaptation and five other mutations as potential virulence determinants [11]. Previously, the same team also reported clinical signs of postweaning multisystemic wasting syndrome in piglets co-infected with porcine circovirus type 2 and a PRRSV strain close to the VP-046 Bis [20]. In a subsequent experimental study, they were able to show that this MLV1-like strain had a higher level of virulence than the parental vaccine strain [20]. In the Eclercy’s study [19], the vaccine-derived strain (PRRS-FR-2014-80-34-1, GenBank ID MN604234) was closer to the VP-046 Bis vaccine strain (99,54% identity). Interestingly, 17 mutations (inducing 12 amino acids changes) were shared between our case farm strain and this previously described VP-046 Bis derived strain, suggesting the involvement of these mutations in pig re-adaptation and/or reversion to virulence. From the data of our study, we can establish a temporal relationship between the detection of the MLV1-derived strain and the onset of reproductive failure. However, a causal relationship has yet to be established and would require experimental infection of specific pathogen free pigs.

In our case, we used different sequencing tools to identify the virus strain recovered from clinical samples. First, ORF5 and ORF7 sequencings were performed to differentiate the vaccine strain used in the herd from the strain isolated on diseased animals. On the basis of this sequencing, we determined a strong proximity between the farm strain and the VP-046 Bis vaccine strain which was never used in the farm. However, because the ORF5 and ORF7 genes represent a very small fraction of the PRRSV genome (4% and 2.4%, respectively), we then performed whole genome sequencing to fully characterize the virus. The full genome sequencing of the PRRSV-1 strain ruled out the presence of a recombinant strain with a MLV commercialized in France or with some known field strains and confirmed the very close proximity to the VP-046 Bis vaccine strain. While in our case whole genome sequencing simply confirmed the results of ORF5 and ORF7 sequencings, in other cases this sequencing approach can detect recombinant strains that could not be detected with ORF5 and ORF7 sequencings alone [21].

We were unable to identify with certainty the source of contamination of the breeding herd. The farm that suffered from the PRRSV outbreak is situated at around 400 m from a post-weaning and fattening farm belonging to another farmer. The two herds did not share personnel or materials. The breeding farm has also a strict control of rodents and the outbreak occurred in January, period with a very low pressure of flies and with no slurry pumping and spreading. The whole genome sequence of the strain circulating in this neighbouring farm showed a 99.46% similarity to the strain isolated in our case report, strongly suggesting that the origin of the outbreak was this neighbouring farm. We had the information that, when the new owner bought the farm in 2021, two batches of newly introduced weaned piglets were vaccinated with the VP-046 Bis vaccine strain in January and February 2021 but not afterwards. The piglets introduced in this neighbouring herd came from a PRRS-free breeding source since 2021. It was the reason why the vaccination was stopped. In the absence of a patent biosecurity failure according to the epidemiological investigations and regular follow-up by the vet practice, we finally hypothesized that aerosol contamination was the most likely route of contamination.

The emergence of the VP-046 Bis-derived strain in this farm may raise concerns about the effectiveness of the existing vaccination program. Given the genetic proximity between the DV and the VP-046 Bis vaccine strains, it was anticipated that there would be strong cross-protection. However, it should be noted that the genetic relatedness between PRRSV strains does not necessarily guarantee cross-protection. In addition, the genetic evolution of the farm strain compared to the VP-046 Bis parental vaccine strain may have led to a reduction in cross-protection with the DV strain.

Furthermore, while Porcilis^®^ vaccination did not prevent the introduction of the VP-046 Bis-derived strain, it certainly reduced its impact. Finally, it was also thanks to the reinforcement of vaccination combined with improved biosecurity measures that the circulation of the farm PRRSV strain was brought under control.

Finally, we calculated the losses due to the PRRSV outbreak. On the 17 affected batches, more than 800 piglets were lost which represented almost 0.6 not-weaned-piglet by productive sow. In a recent study, Torrents and al. (2021) showed a decrease of 1.28 weaned piglets during PRRS instability in 35 breeding herds from a large integrated production system in Spain [22]. The lower impact we measured here during the PRRS outbreak could be explained by the herd immunity at the date of the outbreak, the lower virulence of the vaccine virus derived strain or the quick implementation of corrective measures after diagnosis.

### Conclusion

We present here a case of contamination of a pig farm by an apparently non-recombinant strain derived from the VP-046 Bis vaccine strain, associated with reproductive disorders. This case report highlights the risks of reversion and transmission of PRRS vaccine strains and calls for a judicious use of PRRS MLV.

## Data Availability

No datasets were generated or analysed during the current study.

## Abbreviations

AASV: American Association of Swine Veterinarians
BLAST: Basic Local Alignment Search Tool
EWMA: Exponentially Weighted Moving Average
kg: Kilogram
Mac REBEL™: Management Changes to Reduce Exposure to Bacteria and Eliminate Losses
mg: Milligram
MLV: Modified Live Virus vaccine
ORF: Open Reading Frame
PCR: Polymerase Chain Reaction
PRRS: Porcine Reproductive and Respiratory Syndrome
PRRSV: Porcine Reproductive and Respiratory Syndrome Virus
TTBP: Time To Baseline Production
WGS: Whole Genome Sequencing
